# Metabolic Profile of Offspring from Diabetic *Wistar* Rats Treated with *Mentha piperita* (Peppermint)

**DOI:** 10.1155/2011/430237

**Published:** 2011-04-07

**Authors:** Sandra M. Barbalho, Débora C. Damasceno, Ana Paula Machado Spada, Vanessa Sellis da Silva, Karla Aparecida Martuchi, Marie Oshiiwa, Flávia M. V. Farinazzi Machado, Claudemir Gregório Mendes

**Affiliations:** ^1^Department of Biochemistry, Faculty of Medicine, UNIMAR, 17500-021 Marília, SP, Brazil; ^2^Department of Biochemistry, Faculty of Technology and Foods of Marília (FATEC), 17506-000 Marília, SP, Brazil; ^3^Laboratory of Experimental Research of Gynecology and Obstetrics, Medical School, São Paulo University Julio de Mesquita Filho (UNESP-Botucatu), 18607-741 Botucatu, SP, Brazil; ^4^Department of Biochemistry, University of São Paulo (USP), 05508-900 São Paulo, SP, Brazil; ^5^Department of Biochemistry, Methodist Unversity of Piracicaba (UNIMEP), 16400-680 Lins, SP, Brazil; ^6^Department of Nutrition, Frigorificos Friboi S/A, Industrial Park, 116400-000 Lins, SP, Brazil

## Abstract

This study aimed at evaluating glycemia and lipid profile of offspring from diabetic *Wistar* rats treated with *Mentha piperita* (peppermint) juice. Male offspring from nondiabetic dams (control group: 10 animals treated with water and 10 treated with peppermint juice) and from dams with streptozotocin-induced severe diabetes (diabetic group: 10 animals treated with water and 10 treated with peppermint juice) were used. They were treated during 30 days, and, after the treatment period, levels of glycemia, triglycerides, total cholesterol, and fractions were analyzed in the adult phase. The offspring from diabetic dams treated with peppermint showed significantly reduced levels of glucose, cholesterol, LDL-c, and triglycerides and significant increase in HDL-c levels. The use of the *M. piperita* juice has potential as culturally appropriate strategy to aid in the prevention of DM, dyslipidemia, and its complications.

## 1. Introduction

Diabetes mellitus (DM) is considered to be a syndrome associated with disorders in the metabolism of carbohydrates, lipids, and proteins caused by the absolute or relative lack of insulin [1], and it affects approximately 3 to 10% of the women in the gestational phase [2]. In pregnancy, DM can cause serious problems to mothers and their offspring, contributing with approximately 10% of fetal malformations and approximately 40% of neonatal death [2–4]. The lack of serious maternal glycemic control changes fetal metabolism, which leads to higher risk for fetuses to show metabolic alterations, both in the gestational period and throughout their lives [5, 6]. The alterations resulting from maternal diabetes are related to hyperglycemia and fetal hyperinsulinemia, which affect lipid and protein synthesis [7, 8]. Additionally, maternal hyperglycemia stimulates fetal growth (macrosomia) due to the greater availability of glucose in the blood flow and by the regulation of growth factors [5, 8]. High weight at birth is related to the risk for developing insulin resistance, obesity, and DM2 in the future [9].

Studies show that obesity and DM2 are more frequent in children and adolescents whose mothers were diabetic or had gestational DM. Maternal metabolic disorders affect the growth and the metabolism of descendants, leading to diabetogenesis in different phases of the offspring life, including their pregnancies. Hence, the next generation will also be affected, and the same consequences will influence other generations, thus showing the diabetogenic tendency [10–12].

The problems resulting from DM and its complications bring high costs to the Brazilian Health Care System. The search for medical alternatives to reduce costs can be highly valuable for those living below the poverty line. An alternative can be the use of medicinal plants, which have been increasingly used in the treatment of DM and other metabolic syndrome risk factors [13–15].

*Mentha piperita* L (family Labiatae; genus *Mentha*) is one of the ten most widely used plants in Brazil and is commonly used in the treatment of loss of appetite, common cold, bronchitis, fever, nausea, vomiting [16], spasmodic responses [17], and antimicrobial and antioxidant activities [18, 19]. It is also used for culinary purposes. There is evidence that it has a positive effect on glycemia of the laboratory animal [20]. However, there are no reports in the literature concerning its effects on the offspring of diabetic animals treated with the respective plant. Hence, this study aimed at evaluating glycemia and lipid profile of offspring of diabetic *Wistar* rats treated with *Mentha piperita* juice.

## 2. Methods

### 2.1. Parental Generation

The *Wistar* rats used were kept in the vivarium of UNIMEP (Lins Campus) under controlled conditions (12/12-hour light/dark cycle and ambient temperature of 22 ± 2°C, relative humidity of 60 ± 5%, and water and chow *ad libitum*). These animals were treated according to the “Guide to the care and use of experimental animals,” which delineates the principles by the Canadian Council for the care to laboratory animals. The study was initiated after its approval by the Ethics Committee under registration number 2500000764/2007-47.

### 2.2. Diabetogenesis Period: Diabetes Induction

Nondiabetic rats (*n* = 20) weighing approximately 250 g underwent a seven-day adaptation period in the room where the experiment was conducted. Thereafter, randomly selected rats received an intravenous administration (caudal vein) of 40 mg of streptozotocin (STZ)/Kg (STZ, Sigma Chemical Company, St. Louis, Mo, USA) diluted in citrate buffer (0.1 M; pH 4.5). Nondiabetic animals (*n* = 20) received only the vehicle (citrate buffer) in a volume that was equivalent to that of the diabetogenic drug [17, 18]. As an inclusion criterion, rats were considered to be diabetic (*n* = 20) when showing glycemia above 200 mg/dL and nondiabetic (control, *n* = 20) when showing glycemia below 120 mg/dL.

### 2.3. Mating Period

After diabetes confirmation and separation of the two experimental groups, the mating phase was performed at the end of the afternoon. The rats from each group were grouped, four by four, with a normoglycemic male rat. In the next morning, the males were removed, and vaginal smears were then collected. In case spermatozoa were found in the vaginal smears, that was considered to be day 0 of pregnancy [19].

### 2.4. Pregnancy Period

In the mornings of days 0, 7, 14, and 21 of pregnancy, a blood drop was collected from the caudal vein for glycemia measurement by using glucometer *One Touch Ultra*, Johnson and Johnson. Approximately on the day 21 of pregnancy, after birth of offspring, the dams remained in individual cages with their offspring until weaning (21 days).

### 2.5. Offspring: Weaning and Treatment

After weaning, the offspring (60 animals) were kept in collective cages until they reached adulthood. The adult animals, weighing approximately 250 g, were divided into 4 experimental groups (*n* = 15 animals/group): G1: offspring from control dams treated with vehicle (water), G2: offspring from control dams treated with *Mentha piperita *juice, G3: offspring from diabetic dams treated with vehicle, and G4: offspring from diabetic dams treated with *Mentha piperita *juice. 

The animals in groups G2 and G4 received *Mentha piperita* juice at a dose of 0.29 g/kg once a day (at early morning) for 30 consecutive days. The dose administered to the animals was based on 100 g/L, which corresponds to the daily intake of 200 mL of juice by an adult man weighing 70.0 kg (such intake was based on population consultation).

### 2.6. Mentha piperita Juice Preparation

*Mentha piperita *leaves were washed, weighed (100 g/L), and triturated with water in a blender for 7 minutes. The juice was filtered and frozen in an amber flask. Each flask was thawed daily at ambient temperature two hours prior to administration.

### 2.7. Blood Collection and Biochemical Profile Determination

After 30 days of treatment, the animals were anesthetized with sodium pentobarbital (150 mg/kg) and killed. Subsequently, blood samples were collected in order to determine the biochemical profile (total cholesterol, HDL-c, LDL-c, triglycerides, and glucose). Tests were performed according to the methodology proposed by commercial kits: LABTEST (Lagoa Santa, Belo Horizonte, MG) for glycemia, total cholesterol and HDL-c, and triglycerides and WIENER LAB (São Paulo, SP) for LDL-c. The results were interpreted according to criteria established by the American Diabetes Association [1]. 

### 2.8. Statistical Analysis

Data analysis was performed by using Student's *t* test, and the level of significance adopted was *P* value <.05%.

## 3. Results

**Figure 1:**
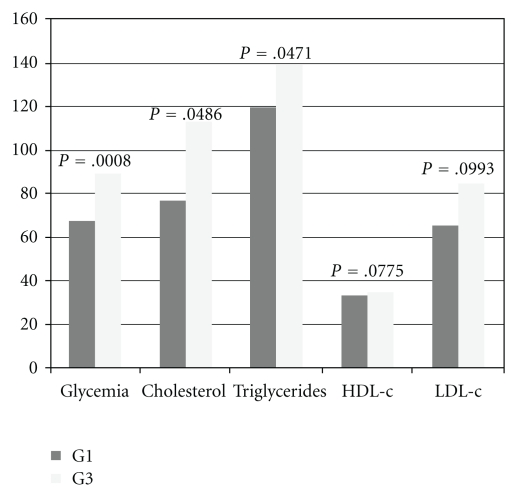
Representation of biochemical profile of G1 and G3.

Figure 1 shows the comparison between the control groups comprising the offspring from diabetic dams and control dams. It was observed that the offspring from group G3 showed significantly higher glycemia, cholesterol, and triglycerides levels when compared to those from group G1. 

**Figure 2:**
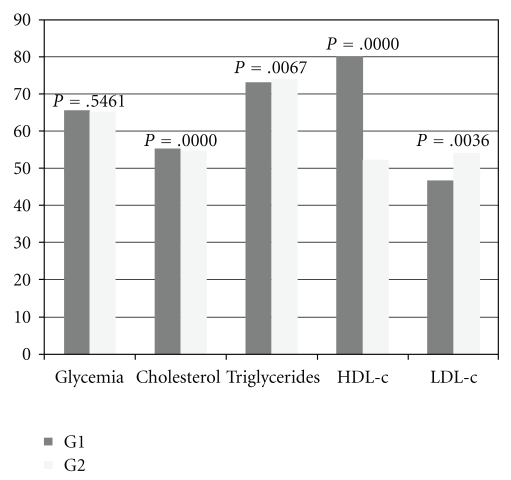
Representation of biochemical profile of G1 and G2.

Figure 2 shows the biochemical profile results for the offspring from control dams (G1) and from nondiabetic dams treated with *M. piperita* (G2). No significant differences were observed for glycemia between these two groups, but there were a significant reduction in plasma levels of cholesterol, triglycerides, and LDL-c and significant increased HDL-c values in the offspring from group G2 in relation to those from group G1.

**Figure 3:**
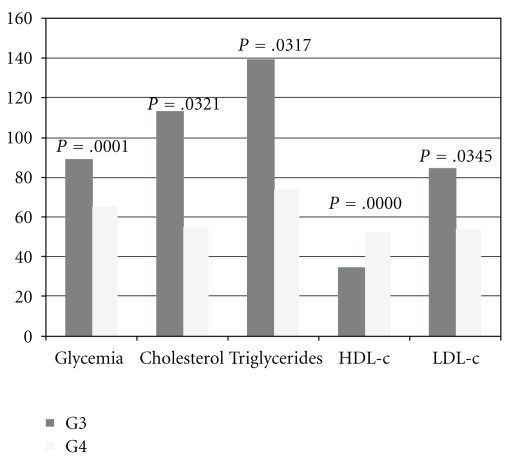
Representation of biochemical profile of G3 and G4.

Figure 3 shows the biochemical profile results for the offspring from diabetic dams treated with vehicle (G3) and from diabetic dams treated with *M. piperita* (G4). The glycemia and the levels of cholesterol, LDL-c, and triglycerides were significantly reduced and the levels of HDL-c were significantly increased in group G4 as compared to group G3. 

**Figure 4:**
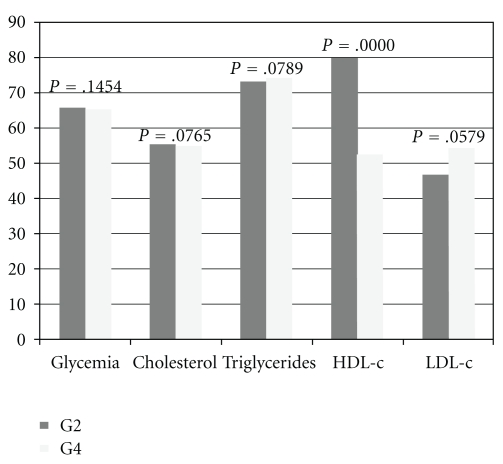
Representation of biochemical profile of G2 and G4.

Figure 4 shows the biochemical profile results for male offspring from nondiabetic dams treated with *M. piperita* juice (G2) and from diabetic dams treated with *M. piperita* juice (G4). There were no significant differences in the glycemia, cholesterol, LDL-c, and triglycerides between the offspring from G4 and G2 groups. It was observed that offspring from G4 dams showed increased HDL-c levels in relation to those from G2. 

**Figure 5:**
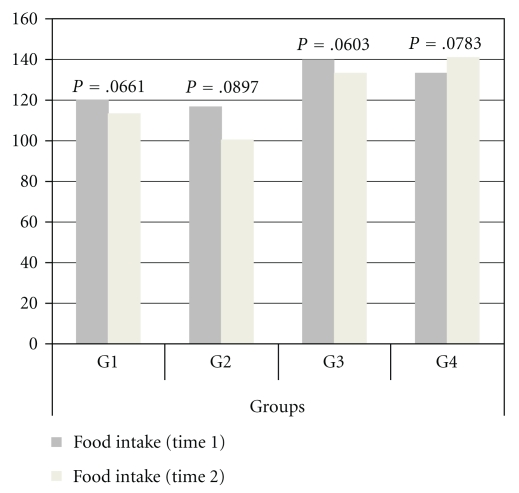
Representation of food intake of G1, G2, G3, and G4.

**Figure 6:**
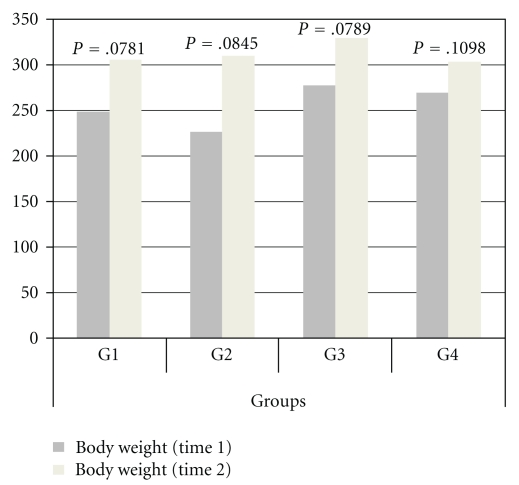
Representation of body weight of G1, G2, G3 and G4.

No differences were found in food intake and body weight among the groups at the beginning and end of the treatment (Figures 5 and 6).

## 4. Discussion

There are a large number of medicinal plants that are popularly used for diabetes mellitus (DM) and hypercholesterolemia treatment [13, 21–24]. Several plants used by the population have been scientifically verified [21, 25–27]. *Mentha piperita* (peppermint) is one of the plants most frequently used by the Brazilian population for therapeutic purposes. Its medicinal use includes anti-inflammatory, antispasmodic, and analgesic activities and the treatment of respiratory and gastrointestinal problems, as well as its antioxidant and antiperoxidant effects [28].

In this study, it was observed that the offspring from diabetic dams showed high levels of blood glucose and lipids. However, significant reductions were also found for the glycemia of offspring from diabetic mothers treated with peppermint juice, thus corroborating the findings by Narendhirakannan et al. [20], who evaluated the effect of *Mentha piperita* on diabetic rats. These results show that the use of peppermint juice can be beneficial in the therapy and/or prevention of DM and its complications in the offspring from dams with diabetes. Other studies in the literature show that the use of plants or antioxidant substances can help avoid damages associated with diabetes [13–15, 29–36]. 

The importance of peppermint can also be extrapolated to the offspring of nondiabetic dams (normoglycemic) as they also benefitted from using the plant. Peppermint juice also positively influences the concentration of plasma lipids in the studied animals, both in the offspring from nondiabetic and in those from diabetic dams. Barbalho et al. [37] observed benefits in glucose and lipid profile after using peppermint juice in normoglycemic Wistar rats. Plants contain a number of biologically active compounds able to modulate lipid and glucose metabolism, of which flavonoids and other antioxidant compounds play a central role for improving the lipid and hyperglycemic profile [38–41]. Visavadiya and Narasimhacharya [35] found hypolipidemic and antioxidant effects in asparagus root. Lee et al. [42] investigated *Wasabia japonica* in vitro and in vivo and observed significant antioxidant activities and anti-hypercholesterolemic effects. Kumar et al. [36] found similar effects after using *Anthocephalus indicus* root.

The species from the *Mentha* genus have substances that may be related to such effects. *M. arvensis* contains menthol, murol, eugenol, thymol, and hydrocarbonates. Menthol and other volatile compounds can be found in the leaves of *M. piperita *[28], and such compounds can be responsible, at least partly, for antioxidant and antiperoxidant effects as observed by Veerapur et al. after using *Ficus racemosa* Stem Bark Extract [43] and Adesegun et al. after using *Phaulopsis fascisepala* [44]. Samarth and Samarth (2009) showed that M. piperita leaf extract possesses high amount of phenolic content, flavonoids content, and flavonols. They also observed that this plant has radioprotective effects possibly because of the amount of phenolic compounds, flavonoids, and flavonols due to their antioxidant and radical scavenging activity [45].

As medicines used to regulate glycemia and dyslipidemia are costly, the use of peppermint juice may be an alternative low-cost strategy to treat noncommunicable diseases associated with the insulin dysfunction. It can also be used to prevent the complications of gestational DM, thus preventing fetal hyperglycemia and hyperinsulinemia, metabolic abnormalities, and the metabolic syndrome in the offspring from mothers with DM. As peppermint also shows antioxidant and antiperoxidant effects, it also can prevent oxidative damages [21, 46–49]. 

Species of *Mentha* are aromatic plants traditionally used as medicinal remedies and culinary herbs but this study suggests that the use of the *M. piperita* juice has potential as a culturally appropriate strategy to aid in the prevention of DM, dyslipidemia, and its complications.

Despite the promising results concerning the use of peppermint, it is fundamentally important to perform further studies in order to evaluate its effects on human beings and the ideal doses to be used.
